# Direct Sensor Orientation of a Land-Based Mobile Mapping System

**DOI:** 10.3390/s110707243

**Published:** 2011-07-18

**Authors:** Jiann-Yeou Rau, Ayman F. Habib, Ana P. Kersting, Kai-Wei Chiang, Ki-In Bang, Yi-Hsing Tseng, Yu-Hua Li

**Affiliations:** 1 Department of Geomatics, National Cheng-Kung University, No.1, University Road, Tainan 701, Taiwan; E-Mails: kwchiang@mail.ncku.edu.tw (K.-W.C.); tseng@mail.ncku.edu.tw (Y.-H.T.); nightterrorz@gmail.com (Y.-H.L.); 2 Department of Geomatics Engineering, 2500 University Drive NW, Calgary, Alberta, AB T2N 1N4, Canada; E-Mails: ahabib@ucalgary.ca (A.F.H.); ana.kersting@ucalgary.ca (A.P.K.); kibang@ucalgary.ca (K.-I.B.)

**Keywords:** Mobile Mapping Systems, direct sensor orientation, camera calibration, direct georeferencing, mounting parameters

## Abstract

A land-based mobile mapping system (MMS) is flexible and useful for the acquisition of road environment geospatial information. It integrates a set of imaging sensors and a position and orientation system (POS). The positioning quality of such systems is highly dependent on the accuracy of the utilized POS. This limitation is the major drawback due to the elevated cost associated with high-end GPS/INS units, particularly the inertial system. The potential accuracy of the direct sensor orientation depends on the architecture and quality of the GPS/INS integration process as well as the validity of the system calibration (*i.e*., calibration of the individual sensors as well as the system mounting parameters). In this paper, a novel single-step procedure using integrated sensor orientation with relative orientation constraint for the estimation of the mounting parameters is introduced. A comparative analysis between the proposed single-step and the traditional two-step procedure is carried out. Moreover, the estimated mounting parameters using the different methods are used in a direct geo-referencing procedure to evaluate their performance and the feasibility of the implemented system. Experimental results show that the proposed system using single-step system calibration method can achieve high 3D positioning accuracy.

## 1. Introduction

Roads are perhaps the most important infrastructures for people’s quality of life. It is used not only for land vehicle transportation, but also for providing the routes for power lines, sewer channels, water supplies, as well as TV and telephone cables. Thus, an efficient and accurate approach for the collection and updating of the road environment information is of extreme importance to the government and public sectors. Traditionally, the acquisition of geographic and attribute information about the road environment, such as traffic signs, road boundaries, sewer manholes, fire hydrants, advertisement boards, and building boundaries, are commonly performed by topographic mapping from large scale aerial photos and/or site surveying. Due to the visual limitations of aerial photos, the demand for site surveying, which is labor intensive and inefficient, is still quite high. Therefore, the development of land-based mobile mapping systems (MMS) has been the focus of several research groups in order to reduce the required manpower and cost while maintaining the necessary accuracy and reliability.

An overview of mobile mapping technology and its applications can be found in [1] and [2]. The MMS is a multi-task system that usually comprises: (i) a platform and power supply, (ii) a control module, (iii) an imaging module, (iv) a positioning and orientation module, and (v) a data processing module. The kinematic platform can be a land vehicle [3], a human operator [4,5], an air vehicle [6], or a marine vehicle [7], either manned or un-manned [8], that provides sufficient power supply for mission operation. The control module is responsible for data acquisition based on time or distance interval. The imaging module could include video cameras, digital cameras, and/or laser scanners. The positioning and orientation module is the most expensive component and crucial for the determination of geographic location of the ground objects. It encompasses a GPS receiver, an inertial measurement unit (IMU), a dead reckoning (DR) system and/or a distance measurement instrument (DMI).

In order to fully explore the potential accuracy of such systems and guarantee accurate multi-sensor integration, a careful system calibration must be carried out [9–13]. System calibration involves individual sensor calibration and the estimation of the mounting parameters relating the system components (e.g., the GPS, IMU, and the imaging sensors). The photogrammetric system calibration, which is the focus of this paper, deals with the camera and the mounting parameters calibration. For multi-camera systems, the mounting parameters encompass two sets of relative orientation parameters (ROPs) [9]: the ROPs among the cameras as well as the lever-arm offsets and boresight angles between the cameras and the navigation sensors (*i.e*., the IMU body frame as the navigation solution usually refers to its coordinate frame). The calibration of the mounting parameters is necessary for directly-oriented multi-camera systems. Since the cameras and the navigation sensors are rigidly mounted on a platform, their geometric relationships are assumed to be invariant. The mounting parameters, which describe their spatial relationships, can be determined using either a two-step or single-step procedure.

The two-step procedure for the estimation of the mounting parameters relating the cameras and the IMU body frame is based on comparing the cameras’ exterior orientation parameters (EOPs), which are determined through a conventional bundle adjustment (indirect geo-referencing) procedure, with the GPS/INS derived position and orientation information of the platform at the moments of exposure. Similarly, the estimation of the ROPs among the cameras can be established by comparing the cameras’ EOPs determined through an indirect geo-referencing procedure. Although such procedures are easy to implement, its reliability is highly dependent on the imaging configuration as well as the number and distribution of tie and control points since these factors control the accuracy of the estimated EOPs.

The single-step procedure, on the other hand, incorporates the system mounting parameters and the POS-based information in the bundle adjustment procedure. The commonly used single-step approach to determine the system mounting parameters is based on the expansion of traditional bundle adjustment procedures with constraint equations [9,10,14]. Such constraints are used to enforce the invariant geometric relationship among the sensors. The drawback of incorporating these constraints to enforce consistent ROPs among the sensors is the associated complicated procedure for doing that, e.g., extensive partial derivatives as well as manual formatting of the camera pairs to be utilized in the relative orientation constraints (ROC). These complexities are intensified as the number of cameras onboard gets larger.

In this paper, a novel single-step procedure, which is more suitable for multi-camera systems, is introduced. The proposed method utilizes the concept of modified collinearity equations, which has already been used by some authors in integrated sensor orientation (ISO) procedures involving single camera systems [5,11,12]. In contrast to the commonly-used constraint equations in previous work, the proposed method is much simpler. The simplicity of the proposed procedure is not affected by the number of the involved cameras and the number of utilized epochs. The proposed multi-camera single-step procedure has the flexibility to be used either for GPS/INS or GPS-assisted photogrammetric systems as well as indirect geo-referencing procedures. Moreover, besides the estimation of the ROPs between the cameras and the IMU body frame, the implemented single-step procedure can also be used to estimate the ROPs among the cameras while enforcing their invariant geometric relationship when GPS/INS data is not available.

This paper starts by outlining the architecture of the designed medium-cost land-based MMS. Then, a discussion of the photogrammetric system calibration is presented. First, the procedure for calibrating the cameras is described followed by a discussion of the proposed mounting parameters calibration. Experimental results are presented next to test the feasibility of the proposed photogrammetric system calibration and to test the performance of the designed system. Finally, the paper presents some conclusions and recommendations for future work.

## 2. System Architectures

**Figure 1.**
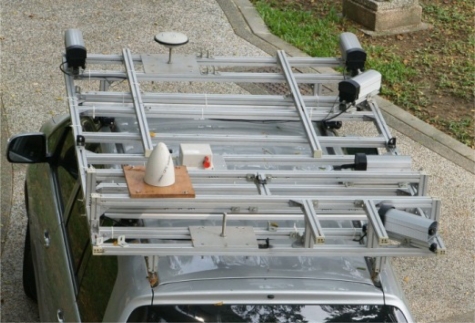
The proposed mobile mapping system.

For the proposed land-based MMS, a reinforced aluminum frame is designed and fixed on top of a van (Figure 1). For the purposes of stereo-measurement, five industrial CCD digital cameras are fixed at the border of the aluminum frame. Two cameras are located in the front (pointing towards the driving direction) for the collection of traffic signs and road surface information. The other three cameras are installed at the right-hand side with pointing angles of 45°, 90° and 135° relative to the driving direction for the collection of road-side features (e.g., building’s façades and advertisement boards). Stereo-measurements can be carried out in imagery captured at the same or different epochs. The GPS antenna is installed at the front while the IMU is located at the middle of all sensors. A detailed description of the major components of the proposed MMS system is discussed in the following subsections.

### 2.1. Position and Orientation System

Since a medium-cost land-based MMS is required in this research, a tactical grade MEMS GPS/INS integrated POS system is adopted (C-MIGITS© III from BEI SDID and a NovAtel© ProPak-V3 GPS receiver). The positional accuracy of such POS system after post-processing, in case of no GPS outage and using kinematic GPS data collection, is about 10 cm for the horizontal direction and 15 cm for the vertical, and the accuracy of the integrated GPS/INS attitude is 0.05° for the roll and pitch and 0.1° for the heading [15], which is satisfactory for many applications [16] and its cost is quite low when compared to a strategic grade IMU and the Applanix© POS AV 510, for example.

This research adopts DGPS positioning with a base station and a tightly-coupled scheme that integrates the IMU and GPS measurements to provide a seamless POS-based solution [15] for direct sensor orientation. The idea is to overcome the disadvantages of the conventional loosely-coupled system. When the number of GPS satellites is less than four, a tightly-coupled scheme is still able to provide integrated navigation solutions through the GPS measurements update. The adopted scheme is particularly suitable for a congested urban environment where GPS signal is frequently obscured.

### 2.2. Digital Imaging Sensors

Three Basler Scouts and two AVT Stingray CCD digital cameras are installed in the proposed MMS. The specifications for these two types of cameras are similar; they both have a pixel size of 4.4 μm and an array dimension of 1,624 × 1,234 pixels. However, the used lenses have different focal lengths, *i.e*., 6.18 mm and 4.87 mm, which result in 60.0° and 72.4° Angular Field of View (AFOV), respectively. Since the AVT Stingray cameras have wider AFOV, they are installed in the front of the car. The cameras are equipped with an electronic shutter that is suitable for extended operation at high image acquisition frequency. The digital interface for the cameras is IEEE 1394b and the frame rate can be as high as 14 fps, which supports high-speed mapping. For example, when driving on a high way at a speed of 100 Km/h and using a 5 fps frame rate, the image acquisition distance interval can be less than 6 m.

### 2.3. Time Synchronization Sensor

For a multi-sensor mobile mapping system, synchronization errors among the sensors will introduce significant position and attitude errors [3]. Generally, the IMU, GPS, and the digital cameras work independently and acquire data at different frequencies. In order to estimate the correct moment of exposure for the digital cameras, a standard timing frame should be established. For that purpose, a Meinberg GPS170PCI timing board is utilized. The timing board has a dedicated GPS antenna (the semi-elliptical shaped antenna in Figure 1). It can record time tags in the GPS time frame with a resolution of 100 ns. Such resolution is precise enough for a land-based MMS considering its operational speed. Since the GPS and IMU measurements are recorded based on the GPS time frame, when the DR sensor sends a trigger pulse to the digital cameras and the timing board at the same time, these sensors can be synchronized. The GPS/INS derived position and orientation can be then interpolated at the moments of exposure. The DR is a dedicated embedded system to estimate the traveled distance and used to send trigger events at a predefined distance interval to the digital cameras and the timing board concurrently. Depending on the vehicle speed, the distance interval can be setup as small as one meter and as large as ninety-nine meters.

## 3. Direct Sensor Orientation

Direct sensor orientation can be performed in two different ways: (i) integrated sensor orientation (ISO) and (ii) direct geo-referencing [13]. In the ISO, the GPS/INS derived position and attitude information are used as prior information in the bundle adjustment procedure together with the image coordinates of tie points. This simultaneous adjustment allows for further improvement in the EOPs and can be performed with or without ground control points. Also, in the ISO procedure, the system mounting parameters can be estimated if appropriate data acquisition and ground control configurations are available. In the direct geo-referencing, on the other hand, the object space coordinates of the image points are obtained from a space intersection procedure using the GPS/INS position and orientation information as well as the system mounting parameters.

There are several factors that might affect the performance of the direct sensor orientation. For instance, the quality of photogrammetric system calibration (*i.e*., camera and mounting parameters calibration), the GPS data quality (which is mainly dependent on the distance from the base station, satellite geometry, and continuity of the GPS lock), the type of the IMU system used, and the quality of the GPS/INS integration process. Investigations into the performance of GPS/INS-assisted photogrammetric systems have demonstrated that the accuracy of direct sensor orientation is mainly limited by the quality of the GPS/INS derived position and orientation as well as the quality of the photogrammetric system calibration. The photogrammetric system calibration, as already mentioned, comprises the cameras and the mounting parameters calibration, which is the focus of the discussion in the following subsections.

### 3.1. Camera Calibration

**Figure 2.**
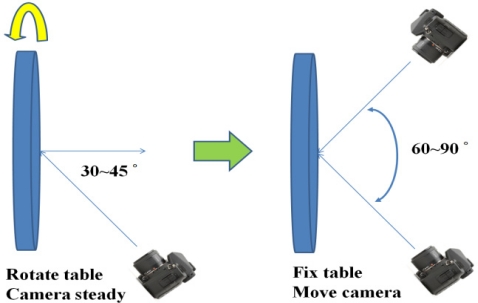
Image acquisition scheme for camera calibration.

The purpose of camera calibration is to mathematically describe the internal geometry of the imaging system, particularly after the light ray passes through the camera’s perspective center. In order to determine such internal characteristics, a self-calibrating bundle adjustment with additional parameters is performed [17]. In this research, we utilize the Photometrix Australis© software package that can automatically recognize and measure the image coordinates of retro-reflective coded targets. Based on this functionality, we developed a rotatable round table, with a radius of 1.2 m, where 112 pillars with heights varying from 10 to 40 cm are established. Then, coded targets are fixed on top of the pillars and the table surface. Instead of changing the camera’s location when acquiring the images, the table is rotated. Moreover, the camera’s viewing direction is inclined at 30°–45° with respect to the table’s surface normal (Figure 2). The round table is rotated with 45° intervals while capturing the calibration images. This results in 8 to 10 images with 60° to 90° convergent angle, which is a strong imaging geometry. In order to decouple the interior orientation parameters (IOPs) and the EOPs during the least squares adjustment, additional 8 to 10 images are acquired with the camera rotated to portrait orientation. Finally, two additional images (landscape and portrait) are taken with the camera’s optical axis perpendicular to the table surface.

Equations (1) and (2) depict the collinearity equations which mathematically describe the light ray from a ground point (A) through the camera perspective center to the image point (a) including the displacements caused by various distortions:

(1) $$x_{\text{a}}=x_{\text{p}}-\text{c}\frac{{\text{r}}_{11}({\text{X}}_{\text{A}}-{\text{X}}_{\text{O}})+{\text{r}}_{21}({\text{Y}}_{\text{A}}-{\text{Y}}_{\text{O}})+{\text{r}}_{31}({\text{Z}}_{\text{A}}-{\text{Z}}_{\text{O}})}{{\text{r}}_{13}({\text{X}}_{\text{A}}-{\text{X}}_{\text{O}})+{\text{r}}_{23}({\text{Y}}_{\text{A}}-{\text{Y}}_{\text{O}})+{\text{r}}_{33}({\text{Z}}_{\text{A}}-{\text{Z}}_{\text{O}})}+\Delta x$$

(2) $$y_{\text{a}}=y_{\text{p}}-\text{c}\frac{{\text{r}}_{12}({\text{X}}_{\text{A}}-{\text{X}}_{\text{O}})+{\text{r}}_{22}({\text{Y}}_{\text{A}}-{\text{Y}}_{\text{O}})+{\text{r}}_{32}({\text{Z}}_{\text{A}}-{\text{Z}}_{\text{O}})}{{\text{r}}_{13}({\text{X}}_{\text{A}}-{\text{X}}_{\text{O}})+{\text{r}}_{23}({\text{Y}}_{\text{A}}-{\text{Y}}_{\text{O}})+{\text{r}}_{33}({\text{Z}}_{\text{A}}-{\text{Z}}_{\text{O}})}+\Delta y$$

In Equations (1) and (2), (X_O_,Y_O_,Z_O_) are the coordinates of the camera’s perspective center, (X_A_,Y_A_,Z_A_) are the coordinates of the ground point (A), (x_p_,y_p_) are the principal point coordinates, c is the camera’s principal distance, and (x_a_,y_a_) are the image coordinates of (a). The camera’s attitude parameters (ω, ϕ, k) are embedded in the rotation matrix elements (r_11_∼r_33_) Finally, Δx and Δy are the image coordinates displacements introduced by the distortions. The mathematical model of the distortions is introduced in Equations (3) and (4). The adopted additional parameters encompass the radial lens distortion coefficients (K_1_,K,_2_K_3_), the de-centering lens distortion coefficients (P_1_,P_2_), and in-plane (differential scale and non-orthogonality) distortion coefficients (b_1_,b_2_). The out-of-plane (image plane un-flatness) distortion is not significant for digital cameras; thus, they are ignored in the adopted camera distortion model [17]:

(3) $$\Delta x=({\text{K}}_{1}{\text{r}}^{2}+{\text{K}}_{2}{\text{r}}^{4}+{\text{K}}_{3}{\text{r}}^{6})\bar{x}+{\text{P}}_{1}({\text{r}}^{2}+2{\bar{x}}^{2})+2{\text{P}}_{2}\bar{x}\bar{y}+{\text{b}}_{1}\bar{x}+{\text{b}}_{2}\bar{y}$$

(4) $$\Delta y=({\text{K}}_{1}{\text{r}}^{2}+{\text{K}}_{2}{\text{r}}^{4}+{\text{K}}_{3}{\text{r}}^{6})\bar{y}+2{\text{P}}_{1}\bar{x}\bar{y}+{\text{P}}_{2}({\text{r}}^{2}+2{\bar{y}}^{2})$$

where x̄ = (x_a_–x_p_), ȳ = (x_a_–y_p_), and $\text{r}=\sqrt{{\bar{\text{x}}}^{2}+{\bar{\text{y}}}^{2}}$. There are two approaches for the determination of the most significant additional parameters. The first one is based on adding one parameter at a time while checking the square root of the a-posteriori variance factor (σ_0_) value, which is a measure of the quality of fit between the observed image coordinates and the predicted image coordinates using the estimated parameters (*i.e*., image residuals). If σ_0_ is reduced significantly, for example more than 0.1 pixels—the expected accuracy of image coordinate measurement, the added parameter is considered significant. Otherwise, the added parameter can be ignored. The second approach is based on checking the correlation coefficient among the parameters and the ratio between the estimated value and its standard deviation (σ), namely significance index. If two additional parameters have high correlation coefficient, e.g., more than 0.9, then the one having the smallest significance index can be ignored. However, if the smallest significance index is larger than a pre-specified threshold, the parameter can still be considered significant. The threshold for the significance index is determined experimentally based on the results from the first approach.

### 3.2. Mounting Parameters Calibration

As already mentioned, in multi-camera systems, the mounting parameters comprise two sets of ROPs: the ROPs among the cameras as well as the ROPs between the cameras and the navigation sensors. There are two main approaches for the determination of such parameters: two-step and single-step procedures. The proposed single-step procedure in this paper, which can be used for the estimation of the two sets of ROPs, and the traditional two-step procedures are explained in the following subsections.

#### 3.2.1. Single-step Mounting Parameters Calibration

The single-step estimation of the lever-arm offsets and boresight angles (*i.e*., ROPs) of the cameras w.r.t. (with respect to) the IMU body frame is performed through an ISO procedure. The incorporation of the GPS/INS position and orientation information as well as the mounting parameters in the ISO procedure can be done by adding relative orientation constraints (ROC) among the cameras and the IMU body frame or by directly incorporating them in the collinearity equations. The latter method has been already used for single-camera systems and has been adapted in this research for use in systems composed of several synchronized cameras since it is the most appropriate solution and allows for easier implementation. The mathematical model used in such method is shown in Equation (5):

(5) $${\text{r}}_{\text{J}}^{\text{M}}=\left[\begin{array}{l} X_{M}\\ Y_{M}\\ Z_{M} \end{array}\right]={\text{r}}_{\text{b}}^{\text{M}}(\text{t})+{\text{R}}_{\text{b}}^{\text{M}}(\text{t}){\text{r}}_{\text{ci}}^{\text{b}}+{\text{R}}_{\text{b}}^{\text{M}}(\text{t}){\text{R}}_{\text{ci}}^{\text{b}}\mu_{\text{j}}^{\text{J}}{\text{x}}_{\text{j}}^{\text{ci}}$$

where:

${\text{r}}_{\text{J}}^{\text{M}}$: is the position vector of an object point (J) relative to a local mapping frame (M);
${\text{r}}_{\text{b}}^{\text{M}}(\text{t})$: is the vector from the origin of the local mapping frame to the origin of the IMU body frame (b) at a given time (t);
${\text{R}}_{\text{b}}^{\text{M}}(\text{t})$: is the rotation matrix relating the local mapping frame and the IMU body frame (derived through the GPS/INS integration process) at time (t) defined by (ω, ϕ, κ);
${\text{r}}_{\text{ci}}^{\text{b}}$: is the lever-arm offset vector (ΔX, ΔY, ΔZ) between the IMU body frame and the *i^th^* camera (ci) perspective center, defined relative to the IMU body frame;
${\text{R}}_{\text{ci}}^{\text{b}}$: is the rotation matrix relating the IMU and the *i^th^* camera coordinate systems, defined by the boresight angles (Δω, Δϕ, Δκ);
${\text{x}}_{\text{j}}^{\text{ci}}=\left[\begin{array}{c} x_{j}^{\mathrm{ci}}-x_{p}^{\mathrm{ci}}-\Delta x^{\mathrm{ci}}\\ y_{j}^{\mathrm{ci}}-y_{p}^{\mathrm{ci}}-\Delta y^{\mathrm{ci}}\\ -c^{\mathrm{ci}} \end{array}\right]$: is the vector from the perspective center to the image point (j) with respect to the *i^th^* camera coordinate system. Note that Δ*x^ci^* and Δ*y^ci^* are defined according to Equations (3) and (4), respectively;
$\mu_{\text{j}}^{\text{J}}$: is the scale factor, which is the ratio between the magnitudes of the object vector, *i.e*., the vector connecting the perspective center and the object point (J)—and the image vector, *i.e*., the vector connecting the perspective center to the image point (j). This scale factor can be implicitly determined from overlapping imagery through the bundle adjustment procedure.

By rearranging the terms in Equation (5), *i.e*., moving the term ${\text{x}}_{\text{j}}^{\text{ci}}$ to the left side of the equation, we can get the form in Equation (6). The observation equations in their final form, *i.e*., the modified collinearity equations, are shown in Equation (7). These equations can be obtained by dividing the first two equations in Equation (6) by the third one while moving the terms ( $x_{p}^{\mathrm{ci}}$, $\Delta x^{\mathrm{ci}}$) and ( $y_{p}^{\mathrm{ci}}$, $\Delta y^{\mathrm{ci}}$) to the left side of the equations. One should note that the scale factor ( $\mu_{\text{j}}^{\text{J}}$) is eliminated through the division process. After deriving the linearized equations in Equation (8), the corrections to the approximate values of the unknown parameters (◯) can be derived through Equation (9):

(6) $${\text{x}}_{\text{j}}^{\text{ci}}=\left[\begin{array}{c} x_{j}^{\mathrm{ci}}-x_{p}^{\mathrm{ci}}-\Delta x^{\mathrm{ci}}\\ y_{j}^{\mathrm{ci}}-y_{p}^{\mathrm{ci}}-\Delta y^{\mathrm{ci}}\\ -c^{\mathrm{ci}} \end{array}\right]=\frac{1}{\mu_{j}^{J}}R_{b}^{\mathrm{ci}}(R_{M}^{b}(t)[{\text{r}}_{\text{J}}^{\text{M}}-{\text{r}}_{\text{b}}^{\text{M}}(\text{t})]-{\text{r}}_{\text{ci}}^{\text{b}})=\frac{1}{\mu_{j}^{J}}\left[\begin{array}{l} N_{x}^{\mathrm{ci}}\\ N_{y}^{\mathrm{ci}}\\ D^{\mathrm{ci}} \end{array}\right]$$

(7a) $$x_{j}^{\mathrm{ci}}=x_{p}^{\mathrm{ci}}-c^{\mathrm{ci}}\frac{N_{x}^{\mathrm{ci}}}{D^{\mathrm{ci}}}+\Delta x^{\mathrm{ci}}$$

(7b) $$y_{j}^{\mathrm{ci}}=y_{p}^{\mathrm{ci}}-c^{\mathrm{ci}}\frac{N_{y}^{\mathrm{ci}}}{D^{\mathrm{ci}}}+\Delta y^{\mathrm{ci}}$$

(8) $$\text{y}=\text{Ax}+\text{e}\;\;\;\;\;\;\;\text{e}∼(0,\sum )\;\;\;\;\;\;\;\;\mathrm{where}\;\;\;\sum =\sigma_{0}^{2}P^{-1}$$

where

***y***: is the *n* × *1* vector of differences between the measured and computed observations using the approximate values of the unknown parameters;
***x***: is the *m* × *1* correction vector to the approximate values of the unknown parameters;
*A*: is the *n* × *m* design matrix (*i.e*., partial derivative matrix w.r.t. the unknown parameters); and
***e***: is the *n* × *1* vector of random noise, which is normally distributed with a zero mean and Σ variance-covariance matrix;
$\sigma_{0}^{2}$: is the *a-priori* variance factor;
P: is the *n* × *n* weight matrix of the noise vector.

(9) $$\hat{x}={\left(A^{T}\mathrm{PA}\right)}^{-1}A^{T}P\text{y}=N^{-1}C$$

The ISO is implemented through a general Least Squares Adjustment (LSA) procedure, *i.e*., the involved quantities in the mathematical model can be treated either as unknowns, stochastic variables or error free (constant) parameters. Initially, all the quantities on the right side of Equations (7) are treated as unknowns. In order to treat the GPS/INS derived position ${\text{r}}_{\text{b}}^{\text{M}}(\text{t})$ and orientation (ω,ϕ,κ) and the ground coordinates of control points ( ${\text{r}}_{\text{J}}^{\text{M}}$) as stochastic variables, pseudo observation equations can be added for such parameters. On the other hand, to treat a specific parameter as a constant (e.g., the parameter corresponding to the *i^th^* row of ***x***), zero values are set for all the elements occupying the *i^th^* row and *i^th^* column of the normal matrix (N) in Equation (9), except for the element occupying the *i^th^* diagonal element, which is set as a one. Also, the *i^th^* row of the *C* vector in Equation (9) is set to zero. This implementation allows for the possibility of utilizing the same model for GPS-assisted, GPS/INS- assisted, or indirectly geo-referenced photogrammetric bundle adjustment. In case of GPS-assisted systems, the boresight angles are fixed to zeros, *i.e*., ${\text{R}}_{\text{ci}}^{\text{b}}=\mathrm{Indentity Matrix}$ and ${\text{R}}_{\text{b}}^{\text{M}}(\text{t})$ becomes ${\text{R}}_{\text{ci}}^{\text{M}}(\text{t})$, and ${\text{r}}_{\text{ci}}^{\text{b}}$ becomes the lever-arm offset vector between the GPS antenna phase centre and the *i^th^* camera (*ci*) perspective center, defined relative to the camera (*ci*) coordinate system. In case of a traditional indirect geo-referencing procedure, besides the boresight angles, lever-arm offset vector ( ${\text{r}}_{\text{ci}}^{\text{b}}$) should also be fixed to zero.

Another advantage of the proposed single-step procedure is the possibility of using the same implementation to enforce the Relative Orientation Constraints among the different cameras of a multi-camera system in an indirect geo-referencing procedure (*i.e*., when GPS/INS data is not available). More specifically, one of the cameras can be used as a reference for defining the position and the orientation of the platform, which are considered as unknowns and therefore determined in the bundle adjustment along with the ROPs relating the other cameras to the reference one. In such a case, the terms ${\text{r}}_{\text{b}}^{\text{M}}(\text{t})$ and ${\text{R}}_{\text{b}}^{\text{M}}(\text{t})$ in Equation (5) should be regarded as the position and orientation of the reference camera (cr): ${\text{r}}_{\text{cr}}^{\text{M}}(\text{t})$ and ${\text{R}}_{\text{cr}}^{\text{M}}(\text{t})$, respectively. Similarly, the terms ${\text{r}}_{\text{ci}}^{\text{b}}$ and ${\text{R}}_{\text{ci}}^{\text{b}}$ in Equation (5) should be regarded as the ROPs of the *i^th^* camera (ci) w.r.t. the reference one: ${\text{r}}_{\text{ci}}^{\text{cr}}$ and ${\text{R}}_{\text{ci}}^{\text{cr}}$ respectively, as shown in Equation (10). Such procedure is denoted in this paper as “Indirect Geo-referencing with ROC”, which is a single-step procedure for the estimation of the ROPs among the cameras:

(10) $${\text{r}}_{\text{P}}^{\text{M}}={\text{r}}_{\text{cr}}^{\text{M}}(\text{t})+{\text{R}}_{\text{cr}}^{\text{M}}(\text{t}){\text{r}}_{\text{ci}}^{\text{cr}}+{\text{R}}_{\text{cr}}^{\text{M}}(\text{t}){\text{R}}_{\text{ci}}^{\text{cr}}\mu_{\text{p}}^{\text{p}}{\text{r}}_{\text{p}}^{\text{ci}}$$

In summary, the mounting parameters relating the cameras to the IMU body frame can be directly estimated through the proposed ISO single-step procedure, which utilizes Equation (5) for incorporating the prior GPS/INS position and orientation information in the bundle adjustment. The same procedure can be used in an indirect geo-referencing mode to directly estimate the ROPs among the cameras (*i.e*., the mounting parameters relating a reference camera to the others in the absence of GPS/INS position and orientation information). In this case, the position and the orientation of the reference camera will be treated as unknowns.

#### 3.2.2. Two-Step Mounting Parameters Calibration

The two-step procedure for estimating the lever-arm offsets and boresight angles of the cameras w.r.t. the IMU body frame is based on comparing the GPS/INS derived position and orientation (*i.e*., ${\text{r}}_{\text{b}}^{\text{M}}(\text{t})$ and ${\text{R}}_{\text{b}}^{\text{M}}(\text{t})$) with the cameras’ EOPs (*i.e*., ${\text{r}}_{\text{ci}}^{\text{M}}(\text{t})$ and ${\text{R}}_{\text{ci}}^{\text{M}}(\text{t})$) determined through an independent bundle adjustment (indirect geo-referencing) solution. More specifically, Equations (11) and (12) are utilized to come up with an estimate of the lever-arm offsets ${\text{r}}_{\text{ci}}^{\text{b}}$ and the boresight matrix ${\text{R}}_{\text{ci}}^{\text{b}}$ of the cameras w.r.t. the IMU body frame:

(11) $${\text{r}}_{\text{ci}}^{\text{b}}(\text{t})={\text{R}}_{\text{b}}^{\text{M}-1}(\text{t})({\text{r}}_{\text{ci}}^{\text{M}}(\text{t})-{\text{r}}_{\text{b}}^{\text{M}}(\text{t}))$$

(12) $${\text{R}}_{\text{ci}}^{\text{b}}(\text{t})={\text{R}}_{\text{b}}^{\text{M}-1}(\text{t}){\text{R}}_{\text{ci}}^{\text{M}}(\text{t})$$

Similarly, the ROPs of the cameras w.r.t. a reference camera can be determined by comparing the cameras EOPs (*i.e*., ${\text{r}}_{\text{ci}}^{\text{M}}(\text{t})$ and ${\text{R}}_{\text{ci}}^{\text{M}}(\text{t})$) with the EOPs of the reference one (*i.e*., ${\text{r}}_{\text{cr}}^{\text{M}}(\text{t})$ and ${\text{R}}_{\text{cr}}^{\text{M}}(\text{t})$), which are the outcome from a traditional indirect geo-referencing solution. To come up with an estimate for the ROPs of the cameras w.r.t. the reference one, Equations (13) and (14) can be utilized:

(13) $${\text{r}}_{\text{ci}}^{\text{cr}}(\text{t})={\text{R}}_{\text{cr}}^{\text{M}-1}(\text{t})\left({\text{r}}_{\text{ci}}^{\text{M}}(\text{t})-{\text{r}}_{\text{cr}}^{\text{M}}(\text{t})\right)$$

(14) $${\text{R}}_{\text{ci}}^{\text{cr}}(\text{t})={\text{R}}_{\text{cr}}^{\text{M}-1}{\text{(t)R}}_{\text{ci}}^{\text{M}}(\text{t})$$

An alternative two-step procedure for the estimation of the cameras’ mounting parameters w.r.t. the IMU body frame would be the utilization of the outcome from the indirect geo-referencing with ROC, introduced in the previous section, instead of the EOPs determined through the conventional indirect geo-referencing procedure. More specifically, this alternative procedure would compare the GPS/INS derived position and orientation with the EOPs of the reference camera (*i.e*., ${\text{r}}_{\text{cr}}^{\text{M}}(\text{t})$ and ${\text{R}}_{\text{cr}}^{\text{M}}(\text{t})$), and the ROPs of the other cameras w.r.t. the reference one (*i.e*., ${\text{r}}_{\text{ci}}^{\text{cr}}$ and ${\text{R}}_{\text{ci}}^{\text{cr}}$), as shown in Equations (15) and (16). Since the invariant geometric relationship among the cameras is enforced in the indirect geo-referencing with ROC procedure, it is expected that the quality of the determined EOPs would be higher than the conventional indirect geo-referencing procedure, which in turn would produce better estimate of the mounting parameters relating the IMU body frame to the different cameras:

(15) $${\text{r}}_{\text{ci}}^{\text{b}}(\text{t})={\text{R}}_{\text{b}}^{\text{M}-1}(\text{t})\left({\text{r}}_{\text{cr}}^{\text{M}}(\text{t})+{\text{R}}_{\text{cr}}^{\text{M}}(\text{t}){\text{r}}_{\text{ci}}^{\text{cr}}-{\text{r}}_{\text{b}}^{\text{M}}\text{(t})\right)$$

(16) $${\text{R}}_{\text{ci}}^{\text{b}}(\text{t})={\text{R}}_{\text{b}}^{\text{M}-1}(\text{t}){\text{R}}_{\text{cr}}^{\text{M}}{\text{(t)R}}_{\text{ci}}^{\text{cr}}$$

It should be noted that the derived ROPs in Equations (11–16) are time-dependent since each exposure instance will give an estimate for the ROPs between any of the utilized cameras and the IMU body frame or the reference camera. An averaging process is usually performed to obtain mean values for the mounting parameters as well as their standard deviation. The advantage of the two-step procedure for the estimation of the system mounting parameters is its simplicity, *i.e*., any bundle adjustment software can provide EOP values for the mounting parameters calibration. However, in order to have reliable estimates, the geometric strength of the imaging configuration as well as the number and distribution of ground control points should be carefully established.

#### 3.2.3. Mounting Parameters Calibration: Final Remarks

The mounting parameters for a GPS/INS-assisted multi-camera system refer to two groups of parameters: (1) the ROPs among the different cameras, *i.e*., the lever-arm offsets and the boresight angles relating a reference camera to the other cameras and (2) the lever-arm offsets and boresight angles relating the IMU body frame to the different cameras.

The estimation of the first group of ROPs can be established using either one of the following approaches:

- Using the ISO in an indirect geo-referencing mode, which is denoted as indirect geo-referencing with ROC (10), one can directly derive an estimate of the ROPs among the cameras.
- Using the conventional indirect geo-referencing, one can derive the EOP of the images captured by the different cameras. The derived EOPs are then used to derive time-dependent estimates of the ROPs according to the formulations in Equations (13) and (14).

On the other hand, the estimation of the second group of ROPs can be done using either one of the following approaches.

- Using the ISO with prior GPS/INS position and orientation information—as explained in (5), one can directly derive an estimate of the mounting parameters relating the cameras to the IMU body frame.
- Using the conventional indirect geo-referencing procedure, one can derive the EOPs of the images captured by the different cameras. The derived EOPs are then used to derive time-dependent estimates of the mounting parameters relating the IMU body frame and the different cameras according to the formulations in Equations (11) and (12).
- Using the indirect geo-referencing procedure with ROC—as explained in Equation (10), one can derive the EOPs of the images captured by the reference camera as well as the ROPs relating this camera to the other cameras. These parameters are then used to derive time-dependent estimates of the mounting parameters relating the IMU body frame and the different cameras according to the formulations in Equations (15) and (16).

The experimental results section will provide a comparative analysis of the performance of these different mounting parameters calibration procedures.

### 3.3. Position and Attitude Information in the Mapping Frame

In this study, the utilized CMigit-III IMU has an East-North-Up (ENU) local navigation coordinate system. The GPS/INS integrated position solution refers to the WGS84 latitude, longitude, and ellipsoidal height, while the orientation is provided as navigation angles, *i.e*. roll (r), pitch (p), and yaw (y) angles. These angles describe the rotational relationship between the IMU body frame (b) and the local navigation frame (N_i_) at the corresponding location for a given time, *i.e*., ${\text{R}}_{\text{b}}^{{\text{N}}_{\text{i}}}(\text{t})$. The navigation frame is a dynamic local coordinate system with its origin at the center of inertial sensor axes triad. The rotation matrix ${\text{R}}_{\text{b}}^{{\text{N}}_{\text{i}}}(\text{t})$ is illustrated in Equation (17):

(17) $${\text{R}}_{\text{b}}^{{\text{N}}_{\text{i}}}{(\text{t})=\text{(R(r)R(p)R(y))}}^{\text{T}=}{\left[\begin{array}{c} \left(\begin{array}{ccc} 1 & 0 & 0\\ 0 & \text{cos}(\text{r}) & \text{sin}(\text{r})\\ 0 & -\text{sin}(\text{r}) & \text{cos}(\text{r}) \end{array}\right)\times \left(\begin{array}{ccc} \text{cos}(\text{p}) & 0 & -\text{sin}(\text{p})\\ 0 & 1 & 0\\ \text{sin}(\text{p}) & 0 & \text{cos}(\text{p}) \end{array}\right)\\ \times \left(\begin{array}{ccc} \text{cos}(\text{y}) & \text{sin}(\text{y}) & 0\\ -\text{sin}(\text{y}) & \text{cos}(\text{y}) & 0\\ 0 & 0 & 1 \end{array}\right) \end{array}\right]}^{\text{T}}$$

The rotation matrix relating the IMU body frame and the local navigation frame should be modified to express the rotational relationship between the IMU body frame and the photogrammetric local mapping frame (M-frame) [18,19]. One way to do that would be to transform the former one into the earth-centered earth-fixed (ECEF) frame through a pre-multiplication with a position-dependent rotation matrix, which is defined by the position of the body frame (ϕ_i_, λ_i_) at a given time, ${\text{R}}_{{\text{N}}_{\text{i}}}^{\text{ECEF}}$, as shown in Equation (18):

(18) $${\text{R}}_{{\text{N}}_{\text{i}}}^{\text{ECEF}}{\text{(}\varphi }_{\text{i}}{,\lambda }_{\text{i}}\text{)}={\left(\begin{array}{ccc} -\text{sin}(\varphi_{i})\text{cos}(\lambda_{i}) & -\text{sin}(\varphi_{i})\text{sin}(\lambda_{i}) & \text{cos}(\varphi_{i})\\ -\text{sin}(\lambda_{i}) & \text{cos}(\lambda_{i}) & 0\\ -\text{cos}(\varphi_{i})\text{cos}(\lambda_{i}) & -\text{cos}(\varphi_{i})\text{sin}(\lambda_{i}) & -\text{sin}(\varphi_{i}) \end{array}\right)}^{\text{T}}$$

In Equation (18), ϕ_i_ and λ_i_ are the WGS84 latitude and longitude of the IMU body frame at a given time. Finally, the rotation matrix between the IMU body frame and the ECEF frame is modified to express the rotational relationship between the IMU body frame and the photogrammetric local mapping frame. In this work, the photogrammetric local mapping frame is defined as a topo-centric frame, denoted as N_0_-frame, with its origin defined within the mapped area (ϕ_0_, λ_0_). Therefore, the rotation matrix from the IMU body frame to the photogrammetric local M-frame can be determined by Equation (19):

(19) $${\text{R}}_{\text{b}}^{\text{M}}(\text{t})={\text{R}}_{\text{ECEF}}^{{\text{N}}_{0}}{\text{(}\varphi }_{0}{,\lambda }_{0}{\text{)R}}_{{\text{N}}_{\text{i}}}^{\text{ECEF}}{\text{(}\varphi }_{\text{i}}{,\lambda }_{\text{i}}{\text{)R}}_{\text{b}}^{{\text{N}}_{\text{i}}}(\text{t})$$

In a similar fashion, the ground coordinates of control points have to be transformed from the WGS84 longitude, latitude, and ellipsoidal height to the photogrammetric local mapping frame. After such transformation, the GPS/INS position and orientation information can be utilized together with the ground coordinates of the control points in the ISO procedure.

## 4. Experimental Results

In this section, experimental results are presented to demonstrate the feasibility of the developed medium-cost land-based MMS and test the validity of the proposed photogrammetric system calibration. First, the camera calibration results are reported. Then, a comparative analysis between the two-step procedures and the proposed single-step procedure for the estimation of the mounting parameters is performed. The estimated camera and mounting parameters are incorporated in a direct geo-referencing procedure (space intersection) using an independent dataset to compare the different methods and evaluate the system performance.

### 4.1. Camera Calibration

The calibration process has been conducted according to the described method in Section 3.1. The five digital cameras AVT-0, AVT-1, Basler-2, Basler-3, and Basler-4 are calibrated independently. The average distance from the camera to the center of the round table is about 2.5 m. Each camera has a total of 20 images taken with around 80° convergence angle providing a strong imaging geometry. Table 1 illustrates the quality of the calibration results for the five cameras. In this table, σ_0_ denotes the square root of the a-posteriori variance factor, which is a measure of the magnitude of the image residuals (*i.e*., the quality of fit between the image coordinate measurements and the estimated parameters—including the calibration parameters). The σ_0_ values are quite acceptable and commensurate with the expected automated image-coordinate measurement accuracy using the retro-reflective targets. The relative accuracy in Table 1 corresponds to the target positioning accuracy when considering the dimensions of the target field. For example, a 1:20,000 relative accuracy means that for a target field whose size is 20 m, the positioning accuracy is about 1 mm.

### 4.2. Mounting Parameters Calibration

**Figure 3.**
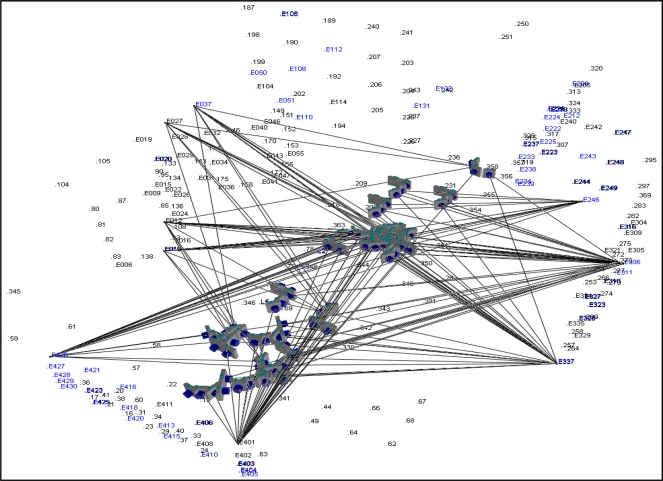
The distribution of acquired images, surveyed targets/control points (in blue), and tie points (in black) together with the intersecting light rays for some of the control points.

The dataset used for the mounting parameters calibration was acquired over an established test field with 67 surveyed targets. Figure 3 is a 3D view illustrating the locations of the surveyed targets/control points (labeled with E prefix) and the acquired images for mounting parameters calibration and validation. For the purpose of demonstrating the imaging geometry, several intersecting light rays from the control points to its corresponding cameras are illustrated in Figure 3. As shown in the figure, the intersection geometry is quite good and the control points are located in different regions of the imagery that are captured from different locations. In this figure, the 3D points without E prefix are the estimated locations for the tie-points. The accuracy of the surveyed points is ±5 cm. The nominal accuracy of the GPS/INS derived position and orientation information is ±10 cm and ±100 s, respectively. A total of 105 images were taken by the 5 cameras at 21 epochs. The first 12 epochs were used for estimating the mounting parameters while the remaining 9 epochs were used for the system evaluation through a direct geo-referencing procedure.

Table 2 presents the estimated ROPs among the cameras, while considering camera “0” (AVT-0) as the reference camera, using the conventional two-step and the proposed single-step procedures. One should note that the GPS/INS position and orientation information is not used in the experiments reported in Table 2. The two-step procedure results were obtained using the derived EOPs from a conventional indirect geo-referencing procedure using Equations (13) and (14). In the single-step procedure (introduced in Section 3.2.1), the indirect geo-referencing with ROC is utilized while considering camera “0” (AVT-0) as the reference camera (*i.e*., the position and the orientation of the platform refers to the position and orientation of camera “0”). A closer look at the reported values in Table 2 reveals a significant reduction in the standard deviations of the estimated parameters when using the indirect geo-referencing with ROC procedure. Such an improvement should be expected since the relative orientation constraint is explicitly enforced in the proposed single-step procedure. It should be noted that the impact of such improvement in the object space would be in the order of 2–3 cm (for an object at a 20 m distance). Therefore, such improvement might not be discerned in the reconstructed object space given that the accuracy of the ground control points is ±5 cm.

Table 3 reports the estimated lever-arm offsets and boresight angles relative to the IMU body frame and the standard deviations from the two-step procedures, *i.e*., the traditional indirect geo-referencing as in Equations (11) and (12) and the indirect geo-referencing procedure with ROC as in Equations (15) and (16)—as well as the single-step procedure. We can observe in Table 3 that the two-step procedures have comparable standard deviations. Considering that the indirect geo-referencing with ROC is expected to yield EOPs with higher accuracy than the conventional indirect geo-referencing, one would expect that the latter would produce mounting parameters with inferior accuracy. This would be the case as long as the GPS/INS position and orientation information has the same level of accuracy as the improved EOPs. However, for this dataset, the improvement in the estimated EOPs when enforcing the ROC is superseded by the inferior GPS/INS accuracy. Also, one should note that the magnitude of the standard deviations of the estimated boresight angles is ranging from ±500 to ±6,000 s, which is an indication that the provided nominal attitude accuracy (*i.e*., ±100 s) is too optimistic.

We can also observe in Table 3 a significant reduction in the standard deviations of the estimated boresight angles when performing the single-step ISO procedure since the invariant relationship among the sensors is explicitly enforced. On the other hand, deterioration in the accuracy of the lever-arm offsets is observed when compared to the ones estimated from the two-step procedures. Here again, this might be attributed to optimistic a-priori accuracy for the attitude angles, which is evident by deterioration in the a-posteriori variance factor (σ_o_)^2^ when comparing the single step procedure with either the traditional two-step procedure or the two-step procedure while enforcing the ROC.

The estimated lever-arm offsets and boresight angles relative to the IMU using the different calibration methods are then used in a direct geo-referencing procedure (space intersection) for an independent dataset (the nine remaining epochs of the acquired dataset) to perform a comparative analysis and to evaluate the performance of the designed system. The direct geo-referencing results (*i.e*., accuracy analysis using 67 check points) are presented in Table 4. A closer look at this table reveals a significant improvement, both in the RMSE, mean and standard deviations for the coordinate differences at the check points, in the intersection results when utilizing the estimated mounting parameters from the proposed ISO (single-step) procedure. On the other hand, the intersection results using the derived mounting parameters from the two-step procedures (*i.e*., using the outcome from the indirect geo-referencing or the indirect geo-referencing while enforcing the ROC) have demonstrated compatible results. Here again, the potential improvement when enforcing the ROC would be more obvious if the accuracy of the GPS/INS position and orientation information is not worse than the improvement gained by enforcing the relative orientation constraints within the indirect geo-referencing procedure.

## 5. Conclusions and Future Works

In this paper, the implementation and accuracy analysis of a medium-cost land-based MMS have been demonstrated. The paper started by outlining the architecture of the proposed MMS. Then, a discussion of the photogrammetric system calibration was presented. First, the procedure for calibrating the cameras was described followed by a discussion of the mounting parameters calibration. A novel single-step procedure for mounting parameters calibration has been presented. The contributions of the proposed method can be summarized as follows: (i) The modified collinearity equations, which have been implemented in previous work for single camera systems only, is expanded in this research work to handle multi-camera systems; (ii) In contrast to the commonly-used additional constraints, the proposed method is much simpler, *i.e*., it does not require extensive partial derivatives as well as manual formatting of the camera pairs to be utilized in the relative orientation constraints (ROC), which might be cumbersome specially when the number of utilized cameras and the number of involved stations get larger; (iii) The proposed implementation can handle either GPS/INS-assisted multi-camera systems, GPS-assisted multi-camera systems, or the traditional indirect geo-referencing procedure; (iv) The introduced method is developed to allow for a single-step estimation of two sets of ROPs (*i.e*., the ROPs among the cameras (when GPS/INS is not available) or the ROPs among the cameras and the IMU body frame), and (v) The proposed procedure will make the calibration process more robust against weaknesses in the geometric image configuration and control distribution (this is achieved by enforcing the relative orientation constraint either explicitly using the ROC or implicitly using the single step ISO procedure). This will have a positive impact on reducing the cost and enhancing the practicality of the calibration process.

Experimental results using real data have shown a significant improvement in the precision of the estimated mounting parameters (especially, the boresight angles) and the object space reconstruction (50 cm reduction in the RMSE values and bias improvement on each axis) when utilizing the proposed single-step procedure. Moreover, the proposed procedure has shown an improved estimation accuracy of the ROPs among the cameras when compared to the estimated ROPs from a two-step procedure. The single-step procedure provides more accurate results for the ROPs among the cameras due to the fact that the relative orientation constraint is explicitly enforced.

Future works will focus on more testing using simulated and real datasets to verify the performance of the proposed system/methods as well as investigating the optimum imaging and control configurations for reliable estimation of the mounting parameters. Also, future implementation will be extended to include previously estimated ROPs among the cameras as prior information when estimating the ROPs between the cameras and the IMU body frame in the developed single-step procedure. In other words, previously estimated relative orientation parameters among the cameras will be included as additional constraints during the single-step estimation of the mounting parameters relating the IMU body frame and involved cameras.

## Figures and Tables

**Table 1.** Quality analysis of the camera calibration.

	**AVT-0**	**AVT-1**	**Basler-2**	**Basler-3**	**Basler-4**
***σ*_0_** (pixels)	0.15	0.16	0.11	0.12	0.11
Relative Accuracy	1:17,600	1:14,400	1:24,100	1:22,900	1:25,800

**Table 2.** Estimated ROPs between camera “0” (AVT-0) and the other cameras.

**Procedure**	**Camera**	**Δω (deg ± s)**	**Δϕ (deg ± s)**	**Δκ (deg ± s)**	**ΔX (m ± m)**	**ΔY (m ± m)**	**ΔZ (m ± m)**
Two-step Indirect	Camera “1” (AVT-1)	0.92777 ±285.5	−0.38012 ±100.1	−2.00209 ±85.2	−0.03 ±0.01	−1.47 ±0.01	0.06 ±0.01
	Camera “2” (Basler-2)	−41.65608 ±144.7	−0.05911 ±140.8	−1.05843 ±198.3	−0.02 ±0.01	−1.49 ±0.01	0.62 ±0.01
	Camera “3” (Basler-3)	−88.95329 ±235.1	1.98176 ±237.6	−0.69070 ±200.0	−0.04 ±0.01	−1.48 ±0.02	1.71 ±0.02
	Camera “4” (Basler-4)	−128.10177 ±321.9	0.52740 ±130.3	−0.33972 ±85.9	−0.06 ±0.01	−1.48 ±0.01	2.47 ±0.01

Single-Step Indirect Geo-ref. with ROC	Camera “1” (AVT-1)	0.93444 ±14.6	−0.40842 ±17.1	−2.00061 ±20.0	−0.03 ±0.0013	−1.48 ±0.0019	0.06 ±0.0014
	Camera “2” (Basler-2)	−41.66469 ±17.5	−0.09493 ±23.3	−1.06639 ±31.4	−0.03 ±0.0017	−1.50 ±0.0022	0.63 ±0.0024
	Camera “3” (Basler-3)	−88.91613 ±25.0	1.95771 ±43.2	−0.69984 ±36.8	−0.04 ±0.0021	−1.49 ±0.0026	1.72 ±0.0031
	Camera “4” (Basler-4)	−128.10779 ±25.1	0.54875 ±52.1	−0.32753 ±38.0	−0.05 ±0.0021	−1.48 ±0.0028	2.47 ±0.0035

**Table 3.** Estimated lever-arm offsets and boresight angles between each camera and the IMU body frame, using different geo-referencing methods.

**Method**	**Camera**	**Δω (deg ± s)**	**Δϕ (deg ± s)**	**Δκ (deg ± s)**	**ΔX (m ± m)**	**ΔY (m ± m)**	**ΔZ (m ± m)**
Two-step (Indirect Georef.) **(σ_o_)^2^**: (0.0025)^2^	Camera “0” (AVT-0)	−0.97284 ±535.7	−0.26904 ±4478.0	1.37450 ±5441.1	0.08 ±0.06	0.49 ±0.02	−1.57 ±0.02
	Camera “1” (AVT-1)	−0.03595 ±698.0	−0.62728 ±4473.9	−0.62241 ±5433.6	0.08 ±0.07	−0.98 ±0.02	−1.49 ±0.02
	Camera “2” (Basler-2)	−42.62160 ±600.5	−1.17411 ±5287.6	−0.21013 ±4784.4	0.09 ±0.06	−0.99 ±0.03	−0.93 ±0.02
	Camera “3” (Basler-3)	−89.92737 ±713.5	0.60325 ±5524.1	−0.93508 ±4580.1	0.06 ±0.04	−0.96 ±0.02	0.16 ±0.02
	Camera “4” (Basler-4)	−129.08182 ±805.1	−0.38674 ±4761.7	−1.40071 ±5167.7	0.04 ±0.04	−0.95 ±0.02	0.92 ±0.02

Two-step (Indirect Geo-ref. with ROC) **(σ_0_)^2^**: (0.0032) ^2^	Camera “0” (AVT-0)	−0.96123 ±658.8	−0.27439 ±4502.9	1.38869 ±5430.5	0.07 ±0.06	0.49 ±0.02	−1.57 ±0.02
	Camera “1” (AVT-1)	−0.01722 ±641.3	−0.65988 ±4491.6	−0.60753 ±5440.2	0.08 ±0.07	−0.98 ±0.02	−1.49 ±0.02
	Camera “2” (Basler-2)	−42.61830 ±658.0	−1.22283 ±5241.0	−0.21120 ±4722.3	0.08 ±0.06	−0.99 ±0.02	−0.92 ±0.02
	Camera “3” (Basler-3)	−89.87900 ±766.2	0.56395 ±5439.1	−0.94801 ±4492.6	0.06 ±0.04	−0.97 ±0.02	0.17 ±0.02
	Camera “4” (Basler-4)	−129.07587 ±762.3	−0.37444 ±4787.0	−1.40052 ±5181.4	0.04 ±0.04	−0.95 ±0.02	0.92 ±0.02

Single-step (ISO) **(σ_0_)^2^**: (0.0077) ^2^	Camera “0” (AVT-0)	−0.90343 ±454.4	0.05174 ±125.7	1.28972 ±119.1	0.07 ±0.12	0.50 ±0.10	−1.55 ±0.10
	Camera “1” (AVT-1)	0.06634 ±454.7	−0.31522 ±125.1	−0.70938 ±120.3	0.08 ±0.12	−0.98 ±0.10	−1.48 ±0.10
	Camera “2” (Basler-2)	−42.53492 ±454.7	−0.92732 ±128.6	0.00765 ±119.3	0.08 ±0.12	−0.99 ±0.10	−0.92 ±0.10
	Camera “3” (Basler-3)	−89.83526 ±455.9	0.55968 ±131.7	−0.53241 ±117.4	0.06 ±0.12	−0.96 ±0.10	0.17 ±0.10
	Camera “4” (Basler-4)	−129.0088 ±456.0	−0.64709 ±129.3	−1.07301 ±119.4	0.05 ±0.12	−0.94 ±0.10	0.93 ±0.10

**Table 4.** Direct geo-referencing RMSE analysis. (unit: m).

**Method**	**RMS-X Mean/Std. Dev.**	**RMS-Y Mean/Std. Dev.**	**RMS-Z Mean/Std. Dev.**	**RMS-TOTAL**
Two-step Indirect	0.71 −0.17 ± 0.69	0.68 0.12 ± 0.67	1.32 0.25 ± 1.31	1.65
Two step Indirect with ROC	0.73 −0.18 ± 0.71	0.70 0.15 ± 0.69	1.36 0.26 ± 1.35	1.70
Single-step ISO	0.47 0.01 ± 0.47	0.60 0.02 ± 0.60	0.80 −0.01 ± 0.80	1.10
